# Integrating capacity development during digital health research: a case study from global health

**DOI:** 10.1080/16549716.2018.1559268

**Published:** 2019-01-16

**Authors:** Adnan A. Hyder, Hannah Selig, Joseph Ali, Elizeus Rutebemberwa, Khaleda Islam, George Pariyo

**Affiliations:** a Milken Institute School of Public Health, George Washington University, Washington, DC, USA; b Department of International Health, Bloomberg School of Public Health, Johns Hopkins University, Baltimore, MD, USA; c Berman Institute of Bioethics, Johns Hopkins University, Baltimore, MD, USA; d Department of Health Policy and Management, Makerere University School of Public Health, Kampala, Uganda; e Institute of Epidemiology, Disease Control and Research, Dhaka, Bangladesh

**Keywords:** Digital health, mHealth, LMICs, capacity development, mobile phone, noncommunicable disease, ESSENCE

## Abstract

**Background**: The Bloomberg Data for Health Initiative Research and Development Arm at Johns Hopkins University Bloomberg School of Public Health, has thus far collected NCD risk factor data from more than 13,000 citizens of three LMICs (Bangladesh, Tanzania and Uganda), and has actively worked to improve capacity with partners worldwide.

**Objective**: This paper focuses on how a research project, can also act as a capacity building activity through its research into collecting non-communicable disease risk factor data using different mobile phone modalities.

**Methods**: This paper evaluates the activities undertaken by the project using the ESSENCE Planning Monitoring and Evaluation Framework for Research Capacity Strengthening.

**Results**: The project was able to successfully integrate meaningful capacity development activities across all partners. Training, networking, sharing resources, joint data collection, and analysis across individual, organizational and project levels were some of the strategies used. The ESSENCE framework allowed a good assessment strategy for this type of work.

**Conclusions**: This paper highlights the value of making capacity development a high priority for digital health research activities, while also considering the need to monitor and evaluate those activities in order for them to be meaningful and sustainable. It also considers how to utilize the ESSENCE Framework to evaluate capacity development activities through research, and how best to adapt the Framework to different programs.

## Background

Low- and Middle-income countries (LMICs) have undergone rapid transitions in the past two decades, with significant shifts economically, demographically and epidemiologically. One of the most significant and ongoing shifts is that of the burden of disease from communicable to non-communicable diseases (NCDs). It is now estimated that 68% of deaths globally are due to non-communicable disease, with more than 80% of these deaths occurring in low- and middle-income countries [1,2]. NCD deaths are only expected to rise in LMICs in the coming years [1,3,4]. Despite this fact, little NCD research is carried out in LMICs, relatively few resources are dedicated to NCDs globally, and interventions are far more likely to be tested in industrialized nations [5]. Concurrent with the changes in the health of global populations, technological advances have altered the everyday lives of billions around the globe. Mobile technologies have rapidly reached saturation in LMICs, from 23 mobile subscriptions per 100 inhabitants in 2005 to 98.7/100 in 2017 [6–8]. Increased mobile phone ownership and use provides a unique opportunity for health professionals to reach and potentially serve several health-related needs of even the most remote populations.

The aims of this paper are to (1) explore how capacity development can be integrated into mHealth research programs and projects, (2) describe the capacity development activities and approaches used thus far in the research and development component of the Bloomberg Data for Health Initiative coordinated by the Johns Hopkins Bloomberg School of Public Health and to (3) describe the initial capacity development outputs of a particular research project – the Bloomberg Data for Health Initiative. This paper will apply the ESSENCE framework [9] to assess the work undertaken thus far by the research and development component of the Bloomberg Data for Health Initiative (BD4HI) and its progress in using mobile phone surveys (MPS) to drive capacity building in a variety of LMIC settings. The following sections will describe this project and its work in the non-communicable disease digital health sphere, present a framework for integrating capacity development into research activities based on previously conducted work and make suggestions for further efforts within this project and elsewhere. It is to be noted that this was primarily a research project, however, given their vital importance, certain capacity development activities were incorporated into the project, and this paper describes a model which can be followed by other programs in a similar position. MPS capacity building in some of these contexts is a novel innovation, and one which must be developed and supported further.

While researchers and funders in recent years have acknowledged the importance of including capacity development activities in all research activities, very little guidance exists on how to make this a reality based on the extant literature [10,11]. While case studies exist, few offers frameworks on how to incorporate these activities or provide any type of evaluation of the value to all collaborators of these activities. Within the field of eHealth/mHealth, even less is available concerning the role of capacity development in research, likely in part because of the recentness of the field and the proliferation of small pilot studies, rather than longer-term partnerships and larger trials [12,13]. Sustainability and scale-up of interventions remain the exception rather than the norm for mHealth projects globally, often leading to frustration within countries at level of overlap between studies and the lack of continuity and collaboration to build capacity and improve interventions [14–16]. Unfortunately, despite being hailed as an intervention that could best suit the needs of countries with low infrastructure and funding levels, capacity to perform mHealth projects independently remains low in many LMICs. More purposeful integration of capacity-building efforts into mHealth research, and more long-term thinking in the design and implementation of these studies, is vital if mHealth is going to fulfil its potential as a tool for health practitioners globally.

## The project

**Figure 1.**
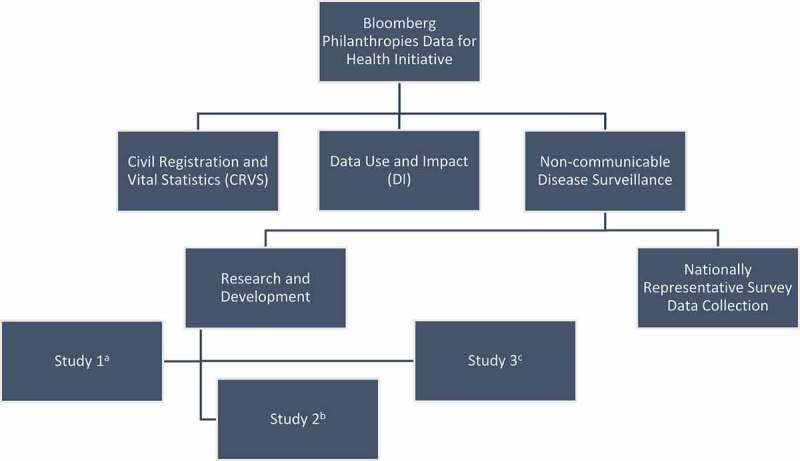
The Bloomberg Data for Health Initiative. (a) Study 1 – evaluation of the impact of incentives and narrative voice on IVR response, contact and completion rates. (b) Study 2 – evaluation of the impact of survey modality (Interactive Voice Response vs Computer-Assisted Telephone Interview) and sampling frame on response and reliability. (c) Study 3 – Evaluation of the Ethical, Legal and Societal Issues surrounding the use of Mobile Phone Surveys for Non-communicable disease surveillance in Low- and Middle-income countries.

The Bloomberg Data for Health Initiative seeks to improve public health data globally in order to empower decision makers and improve the health of populations. To that end, it has chosen to focus on three primary areas – civil registration and vital statistics, data use and impact, and non-communicable disease surveillance (See Figure 1). Within the non-communicable disease component, the Johns Hopkins Bloomberg School of Public Health (JHSPH) has been involved in research and development in order to optimize MPS performance and provide vital information about using these tools in LMICs to policy makers and project implementers globally. JHSPH and country partners have focused their activities on improving the design, response and completion rates of MPS in several LMICs, as well as researching the ethical, legal and societal implications of using these types of surveys in these settings. Please see Table 4 for a full timeline of study activities.

The project has forged and strengthened a number of relationships through its research and development work. In its first phase, JHSPH partnered with Ifakara Health Institute (IHI) in Dar es Salaam, Tanzania; Makerere University School of Public Health (MakSPH) in Kampala, Uganda and Institute of Epidemiology, Disease Control and Research (IEDCR) in Dhaka, Bangladesh. These partner institutions collaborated on producing protocols to guide the research and coordinated all activities within country, including survey translation and recording, Institutional Review Board (IRB) submissions, and qualitative field work to contextualize the mobile phone surveys. Additionally, JHSPH partnered with VOTOmobile (now Viamo), a social enterprise company which operates its own mobile phone survey platform. VOTO was tasked with pushing out IVR surveys in all three selected countries and providing initial data visualization and cleaning for the other partners.

Research and development activities were divided into two phases: formative and optimization. The formative phase focused on qualitative research into previous MPS experience in country, as well as in-country capabilities, needs and the perceptions and attitudes of local community members. The formative phase consisted of Key Informant Interviews, focus group discussions and semi-structured interviews, which included user testing of IVR surveys. This was then followed by an optimization phase in which NCD risk factor surveys were collected via two mobile phone modalities. Further descriptions of these studies can be found in ‘Non-communicable disease risk factors and mobile phones: a proposed research agenda’. A more detailed desciption of study stages and results can be found in Table 1. This paper focusses on the capacity development aspects of the project.

**Table 1.** Bloomberg Data for Health Initiative NCD Research and Development Component Phases.

Phase of Research and development	Example of Activities	Outcomes (examples)
Protocol	Meetings and calls with partners to discuss ideas for protocols Editing of drafts Preparation of appendices and survey transcripts	3 research studies were outlined and written, as were 3 country specific protocols each focusing on different studies
Pre-testing	Determination of platform functionality through sending out small batches of surveys	300 complete surveys were collected in Tanzania, Uganda and Bangladesh
Formative Phase	Key informant interviews, Focus Group Discussions, Semi-structured interviews	45 KIIs, 15 FDGs and 15 SSIs were held in Uganda, Tanzania and Bangladesh
Optimization Phase	Rapid, randomized clinical trials (Microtrials) in which randomly dialed participants are randomly assigned to various arms of trials including incentive amount and incentive structure	7 small randomized controlled trials, with >13000 complete responses were collected in Bangladesh, Tanzania and Uganda
Analysis	Data from microtrials was analyzed Results tabulated Results distributed among the partners	8 publications were submitted for peer review and 8 have been or will be published

## Capacity development activities

The project has conducted a number of activities in the first two years, some of which are also crucial to capacity building efforts. These include trainings prior to both the formative (October 2016–May 2017), and optimization (March–November 2017) phases of work in qualitative and digital health research methods, co-authorship on a published research protocol, with further papers forthcoming, co-ownership of IRB-approved and fully funded research protocols. Routine project activities such as these can serve as capacity strengthening activities when they are carried out in a manner meant to build the collaboration and understanding of all partners.

### Individual

At the individual level of activities, the Johns Hopkins School of Public Health team has strived to build the research capacity of faculty and staff at our various research partner institutions, and to expand their scope and experiences on the modes of research to include digital health. With the training activities undertaken, the goal was always not just successful program implementation, but the preparation of new researchers to carry out a wide variety of research methods with the skillset covered. Trainings were designed not just to train researchers on particular instruments, but in the design and implementation of mHealth studies, and the component pieces therein. Because in-country partners oversaw the day-to-day collection and collation of data it was also vital that they had a strong understanding of the mobile platform as well as the ultimate aims of the project. Trainings were conducted in small group settings, and were largely discussion-based and didactic in nature. This allowed for greater back and forth between teams and the iterative creation and editing of study protocols, materials and surveys to best fit country contexts, and contributed to greater learning for both teams.

Qualitative research training in Bangladesh, Tanzania and Uganda focused on all aspects of successful conduct of key informant interviews, focus group discussions, user-group testing and semi-structured interviews. Training on standard operating procedures for implementing digital health surveys using computer-assisted telephone interviewing (CATI) and interactive voice response (IVR) within the three countries focused not only on the particular research protocols used in the studies, but also helped partner researchers to acquire additional skills necessary to successfully design and implement studies and assess quality of data collected using digital health methods. Implementation training in all three partner countries focused on using the IVR platform to carry out rapid MPS micro trials, and in Bangladesh and Tanzania, managing and conducting CATI surveys with participants selected at random. Partner researchers shared and implemented approaches to optimize survey delivery and response. This has contributed to their developing capacity in digital health research methods, a new and rapidly expanding field of study. Partners in Bangladesh and Tanzania were trained in how to collect data using two different modalities from the same respondent (CATI and IVR) to assess consistency and reliability of survey responses for a range of demographics and NCD risk factors. Such approaches have the potential to contribute information in forming a judgement on the reliability of survey responses from a particular public health survey, contributing to assessments of data quality in the future.

The outputs for these individual-level activities included 54 researchers newly exposed to designing and implementing MPS across the three countries. A further breakdown on the trainings received by researchers can be found in Table 3. Training participants included project coordinators, social scientists, economists, programmers, interns and Principal Investigators across the three partner countries. The research teams at all three partner institutions were closely involved in both program design, country adaptation and implementation, which has expanded global expertise in the harnessing of mobile technology to improve health outcomes. This has also provided vital insights into the specific country context for each partner institution, which is imperative for the successful implementation of any mHealth or eHealth initiative. This new skillset will help collaborating partners in independent creation, deployment and analysis of survey data using mobile technologies.

**Table 2.** Steps in the research process for the Research and Development Component of the Bloomberg Data for Health Initiative and the activities they entail.

Activity	Description	Focus/Content	Frequency
*Trainings*			
Focus Group Discussions training	How to run and manage FDGs	Technical skill development + knowledge	2 (13 researchers trained)
Standard Operating Procedures Training	Presenting finalized research protocols and research plans	Technical skill development + management	2 (10 researchers trained)
Implementation Training	Preparing partners for IVR data collection and analysis	Technical skill development + management	3 (22 researchers trained)
Data collection training	Training for local call-center operatives to perform CATI	Technical skill development	2 (14 researchers trained)
*Products*			
Scientific Papers	Worked with partners to developed manuscripts for publications	Research + analysis Career development	3 co-authorships, 3 independent papers (in progress)
Scientific Proposals	Worked with partners to develop research protocols including all phases of research for submission to IRBs and donor	Technical skill development + Management	3 proposals completed, 3 proposals underway. All submitted have been approved by donor and IRB and funded
Products (reports, conferences etc.)	Drafting of monthly and quarterly reports to donor	Management + Career Development	Monthly, Quarterly and Annual reports
*Research Network*			
Networking	Frequent phone and skype calls, and in-person meetings both in US and at study sites	Management + Career Development	Minimum of monthly meeting/call 11 in-person meetings from 10/16 to 09/17

**Table 3.** Analysis of Bloomberg Data for Health Initiative Capacity Development Activities using Essence Framework.

Capacity Development Level	Activity	Narrative	Output	Research Outcome
Individual	Training for FGDs	Led training of interviewers in qualitative research methods	6 males trained, 7 females trained	15 FDGs completed across 3 countries
	Standard Operating Procedures	Led training of team members in study protocols	3 males trained, 7 females trained	3 research protocols fully implemented, leading to 3 co-authorships in *Journal of Medical Internet Research*
	Implementation Training	Led trainings of team members in study implementation	12 males trained, 10 females trained	>13,000 IVR surveys successfully completed
	Data collection training	Led training of interviewers in CATI techniques and research methods	4 males trained, 10 females trained	800 CATI Interviews successfully completed
Organizational	Papers	Lead authorship and co-authorship of manuscripts detailing research protocols and findings, increased capacity to share research results	Increased data management and analysis capacity Increased ability to prepare manuscripts for publication	All PI are now drafting manuscripts with their teams using project data
	Proposals	Organizational collaboration to improve proposal quality and to adapt to local needs	Meetings held at field sites Conference calls Technical visits	3 funded and completed research studies 3 funded and initiated research studies
	Organizational network building	Building partnerships between various in-country partners including IHI, MakSPH, IEDCR, call-centers, recording studios, and IVR platform owners	Existence of partnership Professional collaboration between entities	Increased collaboration between research institutions and private enterprises involved in research
National and regional systems				
Research network	Forming new partnerships	Through the project JHSPH has reached out to new partners around the world, and sought to build relationships with research institutions globally	Monthly calls and visits to new partners 4 new research networks formed 2 years of partnership – with 2 more planned	3 research protocols developed and implemented 3 research protocols in development 1 webinar

**Table 4.** Project timeline.

	Bangladesh	Tanzania	Uganda
Initial Meetings	July 2015, November 2015	July 2016, August 2016	August 2016
Recruitment of project staff	August-October 2016	August-December 2016	August-November 2016
Field Visits and trainings	October 2016, March 2017, July 2017	August 2016, December 2016, March 2017, June 2017	August 2016, January 2017, August 2017
Qualitative Data collection	March-May 2017	December 2016-April 2017	November 2016-February 2017
Quantitative data collection	May-November 2017	May-August 2017	March-November 2017
US-based training	February 2018	February 2018	February 2018
Final Assessment	March 2019	March 2019	March 2019

As a result of these activities, in-country partners co-authored papers in the *Journal of Medical Internet Research* with JHSPH faculty. One of these papers focused on the research protocol used in data collection, which was written collaboratively by JHSPH, IEDCR, IHI and MakSPH researchers. Locally tailored protocols were developed and approved at all four sites and have since been successfully implemented. Other products include numerous monthly and quarterly reports to donors and other implementing partners; and communications materials including those for social media.

### Organizational

To support this collaborative work, regular meetings were held both virtually and in person beginning in August of 2016. There have been 12 visits to partner countries by the JHSPH team to date, and one meeting of country collaborators at the Hopkins campus, where discussions between partners and JHSPH were held and country leads presented their findings to faculty, staff and students at JHSPH and other US-based partners in a public seminar. While this may be routine program activity, as described above, such activities when carried out well, can serve to help effectively strengthen capacity. Survey design, implementation and data-management capabilities by partners in the realm of digital health has expanded, with increasing expertise in the use of CATI and IVR surveys, and the utilization of those techniques and the accompanying data. JHSPH has helped to build partnerships between our institutional partners and local providers, such as VOTOmobile, local call centers and recording studios, and translators, so that further surveys can be planned and executed independently. Trainings of call center personnel and guidance in the process from initial recording to a full IVR survey, ready for deployment, will lead to self-sufficiency in the production of similar materials in future. The collaborations between all these organizations led to entire teams in each country, from audio recorders to Principal Investigators, being prepared to independently design, implement and report on a variety of health topics beyond NCDs using mobile platforms and modalities; and to incorporate MPS rapidly and effectively into future research or surveillance activities.

Resulting from these activities was the extension of the D4H program in these countries for an additional two years, with three new research studies across four countries being funded and research protocols in the process of IRB submission and approval. In addition to the publication of a special supplement in the *Journal of Medical Internet Research*, many more publications are planned from the qualitative and quantitative data collected, which will be co-authored by Johns Hopkins and in-country researchers. At each collaborating institution, we have established collaborations with a team of translators in the various languages, struck a working relationship with the local audio recording studio, engaged the respondents in NCD research, policy making and implementation, and enhanced capacity to incorporate MPS in our other studies.

### Research network

Through the Data for Health Initiative, JHSPH, MakCHS (Makerere College of Health Sciences), IHI and IEDCR have grown their research networks and built new partnerships nationally and worldwide. More recently, a new collaboration with the *Pontificia Universidad Javeriana* in Colombia was initiated. These partners will continue to learn more about one another’s research and priorities through joint collaborators’ research meetings. Based on experiences and lessons from the project, one webinar has already been held highlighting the lessons learned and the potential for these new digital health technologies to revolutionize surveillance and data collection globally. JHSPH team members have attended multiple meetings and scientific conferences to share the work with other BD4HI study partners, the donor, digital health R&D interested parties, and the larger scientific community. Collaborators in Uganda, Bangladesh, Tanzania and Colombia have engaged with policy makers at the national level to aid in developing fertile ground for translating the results of current and future surveys into practice, and helped to raise the profile of NCDs in a number of LMIC contexts.

## Self-assessment based on essence framework

The recently published Essence Framework was developed by a group of funders whose secretariat is located at the Special Programme for Research and Training in Tropical Diseases (TDR) of the WHO, and is used as a framework to encourage best practices in research capacity strengthening globally [9]. It was designed to evaluate capacity development activities across health fields, and use cases are beginning to emerge in the literature [17–19]. The Essence Framework divides capacity building activities into four primary levels: individual, organizational, national and regional systems and research network. Within each of these levels there are activities, outputs and outcomes [9]. Some outcomes span multiple levels but all focus on increased capacity to conduct and utilize research independently among research partners. In Table 2, work which can be constituted as capacity building by JHSPH and country partners is outlined. A further breakdown based on ESSENCE is provided in Table 3.

The Bloomberg Data for Health Initiative Research and Development arm at JHSPH has completed capacity development activities in three of the four levels outlined in ESSENCE: individual, organizational and research network. Our individual-level activities have resulted in 54 newly trained researchers prepared to collect, manage, analyze and distribute high-quality digital health research data. While their training up to this point has focused primarily on qualitative data, further trainings are planned focusing specifically on quantitative data and its analysis and use. Outputs include improved knowledge about MPS, clarified understandings related to country context, enhanced skills related to conducting MPS, and the incorporation of new knowledge and skills into existing and new projects, both for continuing work with the BD4HI R&D team and independently. Organizationally, project partners have gained experience and expertise in writing papers, mHealth proposals and building their organizational networks to prepare for future mHealth research and in implementing MPS independently. Partnering Principal Investigators are now preparing manuscripts for publication independent of JHSPH researchers, preparing to implement three newly funded and initiated studies and continuing their relationships with private partners which could lead to future independent collaborations on digital health research.

However, little work has been addressed to the national and regional research systems component of capacity building. While acknowledging that this is a weakness of our activities, we were cognizant of our remit which specifically excluded these activities from our arm of the project. Instead, the BD4HI project has a separate branch, the Data Use and Impact component (See Figure 1), which was tasked entirely with engagement activities at the regional and national levels to support uptake of MPS data to bring about policy advancements and long-term changes in the health of populations

Yet even when not in a program’s remit, national-level issues cannot be ignored. Some of the collaborating partners had previous experience with national-level MPS (IEDCR), while this was new territory for others (IHI and MakSPH). Therefore, existing capacity at the national level may vary depending on program site and partner and ought to be explored prior to conducting mHealth research [20]. Because most mHealth research focuses on sub-national or pilot programs, existing capacity at a national and sub-national level should be thoroughly explored before beginning any work and unnecessary duplication avoided whenever possible. Additionally, relevant laws and policies regarding mobile phone use, consent, unsolicited contact and data use and policy must be understood and followed. In this regard, national-level engagement is also important beyond the goal of policy uptake. Under our initiative, we met with and interviewed policy, ethics and MPS experts in all collaborating countries in order to better understand the national environment for these studies, the needs, goals and future directions for mHealth and potential difficulties of MPS in particular contexts. In some cases, this helped raise awareness for MPS at the national level, especially in countries where MPS was not common. It also enhanced our own capacity to navigate potential policy and legal barriers to MPS, such as those relating to random digit dialing (RDD). Additional efforts in involving regional and national level officials and institutions in research and data dissemination will further promote capacity building and sustainability of MPS in LMIC settings.

## Discussion

The ESSENCE framework allows for the evaluation of capacity development activities based on sustainability and long term impact. The particular items of interest in this evaluation include local ownership of activities, sub-national partnerships, enhanced visibility of research institutions, career tracking of researchers, and changes to research, policy, programs and practices. The project has facilitated the formation of sub-national and supra-national partnerships supportive of further future research in MPS and digital health. Local ownership of research was important from the beginning of this project, with extensive in-country input and desire for NCD MPS research and its alignment with in-country research priorities. Joint ownership of research protocols and instruments documents was cemented with the co-authorship of an article describing research activities. Partner institutions led qualitative work and were equal partners for quantitative work, with dual data ownership.

The project also facilitated the connection of research institutions to in-country regulatory agencies and strengthened connections between qualitative and quantitative health researchers. The project led to the establishment of working relationships and partnerships between research institutions, telecommunications regulators, mobile network operators (MNO), locally based call centers and the operators of mobile phone survey platforms, specifically VotoMobile. The establishment of these relationships and partnerships make future independent digital health research for these institutions possible, including the creation of their own unique questionnaires to explore other research areas. The project also increased the visibility of the research institutions partnered with high-impact, cutting edge publications, with both co-authors and primary authors from partner institutions.

### Challenges

While capacity development activities have occurred throughout the planning and implementation of the Research and Development (R&D) Arm of the BD4HI, it is also important to acknowledge the gaps and limitations in the work done thus far. No research project, no matter how integrated its capacity development activities, is a substitute for dedicated programs, fellowships and projects with a sole focus on building capacity. These dedicated programs are able to respond to needs in a far more specific way than research projects and allow for more rapid capacity development. These programs frequently are overlooked in favor of research projects which give greater visibility to high-income country (HIC) researchers. However, dedicated capacity development programs need to be given both greater visibility and greater funding. Particularly in the field of digital health, major gaps exist between the need and interests of countries, and capacity building activities. Continued research activities cannot and will not fulfill needs, and lack of digital capacity will hamper local innovation and solutions. Learning while doing has it role, but does not result in the deep or broad learning necessary to bridge those gaps.

The R&D team from JHSPH has also encountered challenges during the course of its capacity development work, and learned a great deal from colleagues at IEDCR, MakSPH and IHI. Our colleagues were able to provide invaluable insight into research design and questions, and provide the cultural context to explain early findings. The JHSPH was able to gain greater cultural competency from our collaborators, as well as invaluable qualitative experience in different fields. Some of the challenges encountered centered around the ground-breaking nature of the work. Teams from JHSPH had never worked with the in-country call centers or technology partners, or created similar partnerships, prior to this study. Reliance on collaborators and their guidance in forming these partnerships was vital, in the choice of partners and in their makeup. For example, IEDCR has its own call center and performs national CATI surveys, and the JHSPH team leaned heavily on their experience and expertise, learning about the role of the surveyor from IEDCR experts. Their advice has strongly influenced choices regarding narrator for ongoing IVR surveys. The JHSPH team had limited experience with the chosen technology platform before the advent of this study, leading to learning in tandem with our collaborators. Recognizing the value of partnerships that learn together while maintaining reporting relationships under tight donor guidelines was an important growth area for all institutions but especially JHSPH.

Even though we describe the overall experiences there were also some differences in our approach between the three countries based on the context of each site and capacity of our partners. For example, Bangladesh had prior experience in mobile health surveys (such as CATIs) and thus offered a strong platform for this project. Uganda on the other hand had great strengths in community engagement in rural areas and so allowed us to learn from their strategies. Finally, our Tanzanian partner had strengths in qualitative work and we tailored our training program accordingly. The impact of these differences was also more apparent in the implementation of the actual mobile health project rather than just in the capacity development activities.

While capacity building is and should be a goal of all research projects, this component will be more effective the earlier it is planned and input from partners on their capacity development needs and desires is a vital component of that planning. In the first two years of R&D limited discussions with partners on their needs were held. However, for years 3&4 a greater emphasis has been placed on capacity development and the needs of partner institutions. These upcoming activities will include trainings in how to independently design and run mobile surveys using a variety of platforms, exportation and manipulation of data and the creation and running of data visualization platforms for real-time visualization of NCD indicators. Additionally, collaborators are designing costing protocols to measure and predict the cost of conducting large scale mobile phone surveys in country, allowing for estimates to be created for independent grant applications, NGO partnerships, and government institutions. These will aid in influencing governments and other actors to allocate funds in a sustainable manner to use MPS for other surveillance needs.

## Conclusion

The work by the Johns Hopkins University, Makerere University School of Public Health, Ifakara Health Institute and Institute of Epidemiology, Disease Control and Research under the Bloomberg Data for Health Initiative has proven to be an invaluable opportunity to conduct research and collect data in a growing and vital new field. However, all research and development activities should include a capacity building component. Using ESSENCE also provides structure for discussions with partner organizations as to their goals for capacity building through research programs, and their own priorities. Making capacity development a priority in research strengthens all partners and the work conducted. However, capacity development requires constant evaluation and effort to be truly meaningful and sustainable. The ESSENCE framework is a useful tool for organizations, though not all of its criteria may be applicable depending on the particular setting or project, and it must be understood as a template for partners to use as they set their own priorities and agendas. Through partnerships, extensive work in the individual, organizational and research network frames have been carried out, and elevated the capacity of partner organizations to carry out mobile phone research in the future. As the case study shows, purposeful integration of capacity-building efforts into mHealth research is vital if mHealth is going to fulfil its potential as a key solution pathway for global health in this century.

## Paper context

Capacity development should be an integral part of research activities, particularly in the field of digital health. This paper provides a real world example of such explicit inclusion of training and strengthening activities as part of a multi-country mobile health project
